# Roma Socioeconomic Status Has a Higher Impact on Smoking Behaviour than Genetic Susceptibility

**DOI:** 10.3390/ijerph18063206

**Published:** 2021-03-19

**Authors:** Mohammed Merzah, Zsigmond Kósa, János Sándor, Shewaye Natae, Péter Pikó, Róza Ádány, Szilvia Fiatal

**Affiliations:** 1 Department of Public Health and Epidemiology, Faculty of Medicine, University of Debrecen, 4032 Debrecen, Hungary; mohammed.merzah@med.unideb.hu (M.M.); sandor.janos@med.unideb.hu (J.S.); natae.shewaye@med.unideb.hu (S.N.); adany.roza@med.unideb.hu (R.Á.); 2 Doctoral School of Health Sciences, University of Debrecen, 4032 Debrecen, Hungary; 3 Department of Health Methodology and Public Health, Faculty of Health, University of Debrecen, 4400 Nyíregyháza, Hungary; kosa.zsigmond@foh.unideb.hu; 4 MTA-DE Public Health Research Group, University of Debrecen, 4032 Debrecen, Hungary; piko.peter@med.unideb.hu

**Keywords:** smoking behaviours, genetic susceptibility, Roma, socioeconomic status, SNP

## Abstract

It is a matter of speculation whether the high prevalence of smoking among Hungarian Roma (HR) is related to genetic, gene-environmental interactions or cultural factors. Our aim is to compare the genetic susceptibility and possible effects of determinants associated with smoking behaviours in the Hungarian general (HG) and Roma populations. A complex health survey including three pillars (questionnaire, physical and laboratory examinations) was carried out (N_HG_ = 412 and N_HR_ = 402). Risk allele frequencies of ten single-nucleotide polymorphisms (SNPs) were compared, and their combined effect was estimated by computing unweighted and weighted genetic risk scores (GRS, wGRS). The effects of genetic and environmental factors were investigated in regression analyses after confounders were introduced. Socio-economic status (SES) was calculated based on the Kuppuswamy scale 2019. Risk allele frequencies of only four SNPs were found to be different between populations (*p* < 0.01). Median values of GRS did not differ, while the wGRS median was slightly higher among Roma individuals (5.2 vs. 4.9; *p* = 0.02). Roma individuals were more likely to be heavy smokers (OR_males_ = 2.05, 95% CI [1.47–2.86]; OR_females_ = 1.89, 95% CI [1.58–2.25]. Smokers have lower SES compared to never smokers (SES β_HR_ = −0.039, *p* = 0.023; β_HG_ = −0.010, *p* = 0.049). An inverse relationship was found between SES and smoking behaviours (*p* < 0.0001) and was found to be a better predictor of smoking behaviours than genetic susceptibility. Our study findings suggest that the high prevalence of smoking behaviours and nicotine-dependence were not revealed to have a genetic susceptibility among HR individuals; therefore, the highest efforts should be focused on targeting SES-related factors in the Roma population. Strengths of the study: This is the first study carried out to investigate and detect the most relevant factors and the possible genetic background of the extremely high prevalence of smoking based in the Roma population. Limitations of the study: No standard instrument has been used to assess the intensity of addiction to nicotine. Because of some participants’ unwillingness to define themselves as Roma, the overall HR population was not represented by the sample of this study.

## 1. Background

Smoking is one of the main risk factors for non-communicable diseases (NCDs) [1] and is a preventable risk factor for numerous diseases, especially cardiovascular diseases (CVDs) [2]. Smoking behaviour is influenced by genetic and environmental factors and their interactions [3]. Despite the best efforts of global health organizations, which aim to warn populations about the hazards of smoking, its consumption has sharply increased worldwide [4]. The highest rate of tobacco use in 2018 was observed in European countries; the prevalence was 29% among European adults aged more than 15 years, which is estimated to be more than 200 million smokers, among which 35% were women [5]. The prevalence of smoking in Hungary was 28% in the total population, and only less than a quarter of women and a third of men were regular smokers in 2014 based on the Hungarian European Health Interview Survey [6], while the estimated cigarettes smoked per day (CPD) among Hungarians aged ≥ 15 was 23.0 [5]. Of note, the prevalence of smoking differs significantly by age group [7]. Based on a recent epidemiological study carried out in Hungary, the smoking rate was highest among the 35–44 age group (37.5%), followed by the 18–24 age group (26.9%) who smoke on a daily basis. However, occasional smoking was most prevalent in the younger age group [8]. The elderly, aged 55–65 years-old, were on the top list of former smokers and the 45–55 age group were more likely to have ceased smoking [8].

Roma, the largest minority population in Hungary, is the most marginalized ethnicity (estimated population is 8–12 million) in Central-Eastern European countries. They suffer social exclusion, which intensely affects their health outcomes [9]. Higher burdens of disease, low life expectancy, low socioeconomic status, and low education are common among the Roma minority, independent of the county in which they live [10,11,12]. Hungarian Roma (HR) individuals have a higher rate of smoking compared to the Hungarian general (HG) population [11,12]. The prevalence of tobacco consumption by the HR adult population is estimated between 41–72%, which is two to five times higher than in the HG adult population. Remarkably, approximately 51.1% of HR women are smokers and very unlikely to quit; 70.3% of them did not cease smoking during pregnancy [13,14,15]. Furthermore, a study has shown that Roma population were more likely to initiate smoking at early age [16]. A study, conducted on UK individuals aimed to determine the influences of Body Mass Index (BMI) on smoking habits and intensity, suggests that higher adiposity has a strong influence on smoking behaviours and also associated with smoking initiation at younger ages [17]. Among Roma population, a shift from traditional lifestyles characterized by higher caloric intakes and more sedentary lifestyle could lead change in BMI distribution, with obesity becoming significantly more common in the younger Roma population [12,18,19].

Several studies have revealed that genetic characteristics might exist behind the differences in smoking habits/behaviours, and some genetic backgrounds could be exponents towards smoking. Moreover, previous studies provided robust evidence that the high prevalence of smoking behaviours and nicotine dependence was related to genetic susceptibility [20,21,22]. Recent studies have shown that the majority of risk alleles of selected non-communicable diseases have been accumulated in the Roma population [18,23], which support the hypothesis that the health status of Roma is determined by a complex set of inheritable factors.

The smoking prevalence in the Roma population is significantly higher than in the majority population of countries where this has been studied (Croatia, Czech Republic, Hungary, Italy, Lithuania, Macedonia, Slovakia, and Slovenia), even after adjusting for socio-economic factors [10,11,12,13,14,24,25]. If inheritable factors are responsible for differences in smoking patterns of this ethnicity [26], this is the population that one may expect to find it given the high prevalence of smoking and strong nicotine dependence, their distinct ethnic and genetic identity, and high levels of consanguinity [27,28].

In addition to genetic susceptibility, socio-economic status (SES) was found to be a determining factor for smoking habits. The smoking rate is higher among those living in lower SES in developing countries; similarly, in developed countries, smoking rates are 60% higher among those who have experienced different forms of disadvantage than that of among the affluent [29]. Former research revealed that SES is a strong determinant of the health of the Roma population. It was found to be associated with less healthy behaviours such as unhealthful eating, alcohol initiation, and early smoking [13,29].

To improve the health of the Roma minority, conducting studies on selected diseases and health-related behaviours is a priority. The question of whether genetic or environmental factors are more responsible for the higher smoking prevalence in this minority is still open [28]. Hence, this present study was designed to highlight the roles of genetic susceptibility and other possible determinants in the high prevalence of smoking behaviours among the HR population.

## 2. Materials and Methods

### 2.1. Study Design and Population

Formerly, a complex health survey was designed to create a complex database for comparative and association studies to better understand the background of the very unfavourable health of Roma individuals, especially the high burden of cardiometabolic diseases. This cross-sectional study had three main pillars of data (questionnaire-based, physical and laboratory examinations) among the HG and HR adults (20–64 years). Eight-hundred thirty-two participants were recruited from both populations (417 HG and 415 HR) [19]. For the present study, eighteen participants (five HG and 13 HR) were excluded due to missing anthropometric parameters. In addition to creating a complex database, a DNA biobank was also created from the collected data and blood samples.

### 2.2. Patient and Public Involvement

No patient involved.

### 2.3. Statistical Analysis

Plink v.1.9 [30] and Statistical Packages for Social Sciences (SPSS v.25, IBM Corporation: Armonk, NY, USA) [31] were used to analyse the data. To describe the relationships of genotypes with smoking phenotypes (see below) and other demographic variables, a chi-square test was used. Linear regression was applied to predict the best indicator with quantitative smoking behaviours (Model I for CPD; and Model II for age at smoking initiation). To determine the best predictor of smoking status (smoker vs. non-smoker), binary regression models were carried out where (SES), sex, age, BMI, and genetic risk score (GRS) (Model III) or weighted genetic risk score (wGRS) (Model IV) were set as independent variables. Furthermore, multinomial regression analysis was performed to predict smoking behaviours (never smoker, former smoker, moderate smoker, and heavy smoker), as a dependent variable; whereas SES, sex, age, ethnicity, BMI, and GRS (Model V) or wGRS (Model VI) were set as independent variables. A Benjamini-Hochberg multiple correction test was applied to control the false discovery result (FDR) and to compute the adjusted significant results.

### 2.4. Single-Nucleotide Polymorphism (SNPs) Selection

The criteria of SNP selection were described elsewhere [28]. In brief, as a first step, 33 SNPs were selected, based on a literature search on PubMed, as they show a strength effect on smoking behaviours and a consistence pertinent to smoking intensity. All selected SNPs were collected from studies that have a statistically acceptable sample size. Ultimately, only 10 SNPs were included in this present study; the others were excluded for various reasons (unavailability of estimated effect size from genome wide association studies (GWASs) or to avoid multicollinearity based on the fact that only one SNP per linkage disequilibrium (LD) block could be involved).

### 2.5. DNA Isolation and Genotyping

DNA isolation and genotyping method were exposed elsewhere [28]. In brief, DNA preparation was carried out from EDTA-anticoagulation blood samples on the day of sample collection. From plasma samples, DNA were isolated using MagNA Pure LC DNA Isolation Kit–Large Volume (Roche Diagnostics, Basel, Switzerland) according to the manufacturer’s instructions. Genotyping was performed on a MassARRAY platform (Sequenom Inc., San Diego, CA, USA) with iPLEX Gold chemistry by the Mutation Analysis Facility service provider (MAF, Karolinska Institute, Solna, Sweden). Validation, concordance analysis, and quality control were conducted by the Facility according to their protocols.

### 2.6. Genetic Risk Score

Weighted genetic risk scores (wGRS) were calculated based on the risk allele odds ratios of previous studies [20,32,33,34,35,36]. Based on the number of risk alleles carried, each person was assigned a score, where “0” indicated no risk allele, “1” indicated those who were heterozygous for a risk allele, and “2” was assigned to participants who were homozygous for a risk allele. Equation (1) was used for wGRS calculation, where ($W_{OR}$) was derived from the risk coefficients of each risk allele based on the relative effective size determined previously, and ($X_{i}$) was annotated to the number of effective alleles carried by each individual:

(1) $$wGRS=\overset{I}{\underset{i=1}{\sum }}W_{OR}\;X_{i}$$

The unweighted genetic risk score was calculated for each individual based on Equation (2) where $G_{i}$ is the number of risk alleles for each SNP. Then, all scores for each person of the ten SNPs were summed with the assumption that all SNPs have a similar effect:

(2) $$GRS=\overset{I}{\underset{i=1}{\sum }}\;G_{i}$$

### 2.7. Smoking Phenotypes

Qualitative smoking behaviours were classified into four categories. Never smoker (NSM) was defined as a person who smoked ≤100 cigarettes in his/her entire life. Participants who consumed ≤5 CPD or who had smoked more than 100 cigarettes in their lifetime but have been smoke-free for at least one year were defined as former smokers (FSM). Moderate smokers (MSM) were participants who smoked 6 ≤ CPD but <20, while participants who smoked ≥20 CPD were classified as heavy smokers (HSM) [12]. Later during the analysis, the former, moderate, and heavy smokers were combined and categorized as smokers. Furthermore, quantitative smoking behaviours (CPD, and age at smoking initiation) were also included in this study.

### 2.8. Hardy-Weinberg Equilibrium (HWE)

Deviation of the genotype distributions from Hardy-Weinberg Equilibrium (HWE) was inspected for HG and HR population separately. The rs3762611 was excluded from further analysis due to failing to follow HWE (Supplementary Table S1). In addition, rs4105144 could not be genotyped due to poor clustering and was not possible to call correctly according to the MAF service provider.

### 2.9. Socioeconomic Status (SES)

SES was characterized by using the modified Kuppuswamy Scale 2019. The criteria for calculation were described elsewhere [37]. Briefly, SES were categorized into five groups, upper, upper-middle, lower-middle, upper-lower and lower, based on education level, occupation, and monthly family income.

### 2.10. Ethical Approval

The Committee of the Hungarian Scientific Council on Health approved the protocol (61327-2017/EKU). All subjects agreed to participate in this study by signing a written informed consent.

## 3. Results

Descriptive statistics were performed to summarize demographic data of participants. Females of both populations represented 64% of participants; among them, 56.5% were HR individuals. Three-quarters of the study populations were aged <50 years. More than two-thirds of HG individuals were non-smokers, while almost the same proportion of the HR individuals were smokers. A highly significant difference was detected regarding smoking status among the study populations (*p*-value < 0.001). HR males and females were (OR = 1.9 and 2.1, respectively) more likely to be smokers compared to their HG counterparts (Supplementary Table S2). Lower socioeconomic level was reported among HR individuals, which was highly significant (*p*-value < 0.0001) compared to HG individuals. A total of 78.6% of HR individuals were living in the lower to upper-middle SES categories (See Table 1).

Risk allele frequencies among HG and HR populations differed significantly for rs2673931, rs6517442, and rs2235186 at the <0.05 level and for rs578776 at the <0.0001 level (Supplementary Table S3). After adjusting and removing all missing genotypes, there were still no differences in the results found among all significant SNPs. Conversely, some differences were obtained after performing correction tests among males and females separately. The frequencies of rs578776, rs6517442, and rs2235186 differed significantly at a level of *p* < 0.05 among males before adjusting (0.021, 0.036, and 0.015 respectively), while they were insignificant after adjusting. Among females, however, the frequency of rs578776 was highly significant even after multiple correction test (*p*< 0.0001) (Supplementary Tables S4 and S5).

A chi-square test was performed to detect the relationship between genotype and smoking status in the study samples and separately in each population with smoking behaviours. For smoking status, the additive model shows significant relationships for rs16969968-A, rs2036534-T, rs2235186-A, rs578776-G, and rs6517442-C (Table 2). Homozygous and heterozygous statuses for risk alleles for all significant SNPs were highly frequent among HR smokers (Table 2). Regarding smoking behaviours, however, the additive model revealed that no SNP was significant among HR individuals, while the only significant (at *p* < 0.001) result obtained among HG individuals was for the additive model of rs2235186-A with smoking behaviours (Supplementary Tables S6 and S7).

Generally, the average CPD was higher among HR individuals (12 ± 13) than among HG individuals (5 ± 9). Males of both populations consumed more CPD compared to females; however, HR males (14 ± 7) consumed double the CPD compared to HG males (6 ± 5). HG females smoked (4 ± 4) one-third the CPD compared to their counterparts from the HR population (12 ± 5). In addition, CPD was high among dominant homozygous genotypes for rs6517442-C, rs16969968-A, rs2235186-A, rs578776-G, and rs4142041-G alleles. Additive-dominant models for rs10490162-T and rs2673931-T were related to high numbers of cigarettes smoked per day in the whole population (Supplementary Figure S1a,b).

The association of CPD with genotype was varied if we consider study populations separately. Genotypes with risk alleles for some SNPs were highly significantly associated with average CPD only in HG individuals, while others were associated only in HR individuals. In general, HG individuals who carried either homozygous or heterozygous risk alleles of 6 SNPs out of eight had higher smoke rates. In contrast, four SNPs out of eight were interconnected with high smoke rates among HR individuals (Supplementary Figure S2(a1,a2,b1,b2)).

Ages at smoking initiation among HR and HG individuals were not different. The mean ages at smoking initiation were 16 ± 1.774 years and 17 ± 0.308 years for HR and HG populations, respectively. Dominant homozygous and heterozygous at rs10490162-T and rs6517442-C were related with an early age at smoking initiation. However, in the whole study population, only dominant homozygous genotypes at rs16969968-A, rs2235186-A, and rs4142041-G were related to an early age at smoking initiation (Supplementary Figure S3a,b).

Oddly, HR individuals who carried dominant homozygous or heterozygous risk alleles for all SNPs except rs2673931-T began smoking at an early age (Supplementary Figure S4(b1,b2)). Nevertheless, an early age at smoking initiation was related to homozygous risk alleles being carried among HG individuals for six SNPs (Supplementary Figure S4(a1,a2)). Remarkably, HR individuals who carried dominant homozygous alleles at rs6517442 began smoking at an early age (15 ± 0.8) (Supplementary Figure S4(b1)), while a dominant homozygous allele at rs16969968 was linked to an early age of smoking initiation (15.6 ± 0.07) among HG individuals (Supplementary Figure S4(a2)).

The median and interquartile range (IQR) of GRS were equivalent for HR and HG individuals (*median* = 9, *p* = 0.618; IQR = 7–10). However, the wGRS median for HR individuals was slightly higher (*median* = 5.2) than that for HG individuals (*median* = 4.9), with *p* = 0.02. The IQRs of wGRS were 3 to 5 for HG individuals and 4 to 6 for HR individuals (Supplementary Figure S5); consequently, wGRS were right-shifted among HR individuals. Heavy smokers were 1.07 among HR individuals and 1.02 among HG individuals and were more likely to have higher GRS compared to other smokers. The wGRS was higher among heavy smokers of the HG population (OR = 1.34; CI = 0.32–0.97); conversely, lower wGRS among heavy smokers the of HR population were noted compared to other smokers of the same population (OR = 0.94; CI = 0.86–1.42). See Supplementary Table S8.

Different sets of regression models were set up to identify the best predictors behind smoking behaviours. Linear regression (Model I & II) was performed to predict the best factors that might associate with quantitative smoking behaviours (cigarettes per day, CPD, and age at smoking initiation). Model I shows that HR individuals were 33% (*p* < 0.001) more likely to smoke more cigarettes than HG individuals. In relation to age at smoking initiation, HR individuals were more likely to start smoking at an early age (standardized β = −0.23, *p* < 0.001). In addition, a lower BMI was found among people who smoke more and initiate smoking at early ages with respect to the other classifications; however, gender was significantly associated with CPD but not with smoking initiation, (Supplementary Tables S9 and S10).

Smoking status was fixed as a dependent variable in a binary regression analysis, where socioeconomic status (SES), sex, age, BMI, and GRS (for Model III—Table 3A) and wGRS (for Model IV—Table 3B) were set as independent variables. These models show that SES was the best predictor for smoking behaviours, with a significant *p* < 0.05 among populations, separately. Smokers have a lower SES compared to never smokers of both populations (SES β = −0.039, *p* = 0.023 for HR; β = −0.010, *p* = 0.049 for HG). Furthermore, HR smokers were found to have a lower BMI compared to those who are non-smokers (β = −0.150, *p* = 0.004).

Multinomial logistic regression analysis was calculated to predict smoking behaviours based on genetic risk score (GRS) for Model V, weighted genetic risk score (wGRS) for Model VI, and SES, age, BMI, sex and population (HG fixed as a reference category). The regression equation was significant (*p* < 0.0001), with an R^2^ of 0.176. Smoking behaviours were significantly predicted based on socioeconomic status (SES) and population. An inverse relationship was found between SES categories and smoking behaviours, (*p* < 0.0001); moreover, HG individuals were less likely to be smokers than HR individuals (*p* < 0.05) after adjusting for age, BMI, and sex and genetic factors. (Table 4). In sum, SES was found to be a better predictor of smoking behaviours than genetic susceptibility.

## 4. Discussion

People living in segregated areas not only experience social exclusion [26,38], but also genetic susceptibility, which might increase their risk for some unhealthy behaviours or diseases [38,39]. Knowing the genetic characteristics behind smoking behaviours is crucial so that new interventions can be utilized for smoking cessation. To the best of our knowledge, no previous study has been performed on genetic comparisons of the qualitative and quantitative smoking behaviours among Hungarians. A genetic load comparison was performed in our former study, as no load of genetic susceptibility was found related to increased smoking rate in HR individuals, but phenotypic data were not available. Therefore, this study was conducted to demonstrate the genetic background and the predictors behind smoking behaviours among those populations with the availability of phenotypic data. Our study findings suggest that the high prevalence of smoking behaviours and nicotine-dependence were not revealed to have a genetic susceptibility among HR individuals.

Equivalent GRS-medians were found in both populations. Based on the wGRS, HR individuals had slightly higher median-risk scores than HG individuals. Weighted and un-weighted genetic risk scores were not related to smoking behaviours, while ethnicity and socioeconomic status (SES) were significantly linked to smoking behaviours. Although GRSs were equivalent in study populations, heavy smokers still had higher risk scores. This might be due to the selected SNPs, as most of them were related to nicotine dependence, which is higher among heavy smokers [40]. Therefore, further studies including more SNPs related to smoking behaviours are required.

As the HR population lives in segregated areas with economic difficulties, the environmental and cultural factors might be dominant towards smoking habits. In general, tobacco use follows clear socio-demographic patterns and is becoming increasingly concentrated in lower socio-economic group [29,41]. Smokers across Europe were more likely to be unemployed, self-employed or manual workers; the latter were the most prevalent smokers among the employees (40% smoke). Correspondingly, tobacco use has a strong connection with lower levels of educational attainment [29]. A Roma health report, issued by European Union, reveals that these patterns are also reflected in Roma populations, where smoking prevalence is typically high, with smoking initiation at younger age and tendency to smoke more cigarettes than the non-Roma population [29,42].

Moreover, SES proved to be a dominant factor that might influence HR health and lifestyles [13,43,44]. HR individuals living in economic difficulty not only have a higher smoking rate but also a lower chance to quit smoking [15,45,46]. Living in a lower economic status increases the likelihood of being a smoker (Supplementary Table S11); this finding is supported by others [9,13,29,44,46]. Sixty-five percent of HR individuals were smokers, from which 77% were living in lower-middle categories of SES, and from which 50% were heavy smokers. This shows the highly correlated link between smoking behaviours and SES. In other words, the lower the economic status in which the HR individuals live, the higher the prevalence of heavy smoking habits would be. Therefore, categorizing strategies of interventions and disease prevention as alternative implications could be achieved through revealing the effects of segregation. Public health efforts should be dedicated to detecting social/cultural barriers to reducing smoking rates.

It is clear that many policies that have led to the decrease in smoking rate in the general population were less successful in the HR [11]. A qualitative study, conducted in Slovenia, described smoking as a feature of Roma ethnic, cultural, and individual identity since the cigarette introduced by older family members to younger ones, considering it as a part of “growing up” [47]. Hence, qualitative research on understanding the barriers to reduction in smoking rate among HR are urgently needed. It is noteworthy that most Roma individuals reject to support policies related to tobacco-control-measures [11]. However, studying the extent to which Roma populations feel marginalized and discriminated by public authorities must take full account, and messages must be developed through a shared process with Roma participation, in ways that avoid stigmatization.

Heavy smokers were assumed to be nicotine-dependent [39,44,48]; based on that assumption, we considered participants as heavy smokers based on the number of cigarettes smoked per day, which was the major limitation of this study. Instead, the Fagerstrom Test for Nicotine Dependence (FND) [49], the Wisconsin Inventory of Smoking Dependence [50] or the Nicotine Dependence Syndrome Scale [51] would have been useful in our study, as nicotine dependence phenotypes might be measured accurately. Another limitation of this study was that the overall HR population was not represented by the sample of this study. Attributable to some participants’ unwillingness to define themselves as HR, this might contribute to include some HR individuals being classified under the HG group, which might have consequences on some underestimations of genetic susceptibility outcomes.

Altogether, this is primarily a study of the differentiation of genetic characteristics of the qualitative and quantitative smoking behaviours among HG and HR populations. Our hypothesis, which assumed that HR individuals are genetically susceptible to smoking behaviours, was rejected. Therefore, efforts of future studies should be focused on non-genetic factors, especially SES determinants, while even further studies on genetic factors that predispose ethnic minorities to a higher risk of smoking are also needed and might contribute to tobacco control.

## Figures and Tables

**Table 1:** Characteristics of the study populations.

Variables		Population		χ^2^	*p*-Value
		HG (N = 412) *n* (%)	HR (N = 402) *n* (%)		
Sex	Male	186 (45.1)	106 (26.4)	28.699	**<0.0001**
	Female	226 (54.9)	296 (73.6)		
Age (years)	20–29	70 (16.9)	79 (19.3)	9.187	0.102
	30–39	76 (18.4)	81 (19.8)		
	40–49	117 (28.4)	103 (25.1)		
	50–59	91 (22.2)	95 (23.2)		
	≥60	58 (14.1)	44 (10.8)		
Socioeconomic Status (SES)	Lower	0 (0)	21 (5.2)	26.983	**<0.0001**
	Upper lower	69 (16.7)	106 (26.4)		
	Lower middle	175 (42.5)	189 (47.0)		
	Upper middle	150 (36.4)	86 (21.4)		
	Upper	18 (4.4)	0 (0)		
Smoking Status	Smoker	135 (32.8)	262 (65.2)	86.497	**<0.0001**
	Non-smoker	277 (67.2)	140 (34.8)		

Bold font highlights significant differences between populations. HG = Hungarian general; HR = Hungarian Roma.

**Table 2:** Genotype by smoking status in study populations (N = 814).

SNPs	Gene	Genotype	HG (N = 412)		HR (N = 402)		*p*-Value
			SM %	NSM %	SM %	NSM %	
rs10490162-T	*NRXN1*	C C	0.5	1.0	0.8	0.0	0.271
		T C	4.4	14.7	11.0	5.9	
		T T	27.9	51.5	53.2	29.2	
**rs16969968-A**	*CHRNA5*	A A	3.4	7.3	6.4	5.6	**0.031**
		G A	18.8	30.3	28.5	13.7	
		G G	10.5	29.6	30.3	15.5	
**rs2036534-T**	*AGPHD1*	C C	0.2	3.2	4.1	2.3	**0.039**
		C T	9.6	23.8	24.2	12.0	
		T T	22.8	40.4	36.9	20.6	
**rs2235186-A**	*MAOA*	A A	7.1	11.3	15.0	7.4	**<0.0001**
		A G	9.3	16.9	26.4	10.7	
		G G	16.2	39.2	23.9	16.8	
rs2673931-T	*TRPC7*	C C	5.2	8.8	13.2	7.1	0.121
		C T	16.2	31.9	33.0	18.5	
		T T	11.3	26.5	19.0	9.1	
rs4142041-G	*CTNNA3*	A A	13.4	21.8	28.1	15.1	0.300
		A G	15.6	36.1	28.8	15.8	
		G G	4.2	8.9	7.9	4.3	
**rs578776-G**	*CHRNA3*	A A	0.7	5.7	11.6	7.5	**<0.0001**
		G A	13.9	25.4	30.2	12.9	
		G G	18.4	35.8	23.2	14.7	
**rs6517442-C**	*KCNJ6*	C C	3.7	4.9	6.3	5.1	**0.008**
		T C	10.5	31.1	32.2	14.5	
		T T	18.6	31.3	26.6	15.2	

Bold font highlights significant differences. SM = smokers; NSM = non-smokers, HG = Hungarian general; HR = Hungarian Roma. Risk allele is written beside each SNP.

**Table 3:** Association of SES with smoking status by study group.

**A**							
	**Hungarian Roma (*n* = 402)**		**Hungarian General (*n* = 412)**				
	**β**	**95% CI**	***p*-Value**	**β**		**95% CI**	***p*-Value**
SES	−0.039	0.023–0.026	**0.022**	−0.037		0.044–0.064	**0.046**
GRS	−0.003	−0.039–0.034	0.148	0.034		−0.03–0097	0.302
Sex	−0.026	−0.124–0.236	0.609	−0.236		−0.588–0.116	0.198
Age	−0.058	−0.091–0.039	0.267	0.061		−0.048–0.17	0.272
BMI	−0.150	−0.259–0.031	**0.004**	−0.124		−0.314–0.067	0.203
**B**							
	**Hungarian Roma (*n* = 402)**				**Hungarian General (*n* = 412)**		
	**β**	**95% CI**	***p*-Value**	**β**		**95% CI**	***p*-Value**
SES	−0.039	0.052–0.072	**0.023**	−0.010		0.059–0.069	**0.049**
wGRS	−0.163	−0.203–0.088	0.095	0.065		−0.040–0.171	0.157
Sex	−0.026	−0.203–0.631	0.355	−0.263		−0.064–0.077	0.129
Age	−0.058	−0.229–0.068	0.288	0.072		−0.046–0.190	0.231
BMI	−0.150	−0.441–−0.081	**0.004**	−0.150		−0.346–0.046	0.134

Bold font means significant. R^2^ = 0.22 (**A**), R^2^ = 0.23 (**B**); smoking status (smokers and non-smokers) was fixed as a dependent variable. SES= socioeconomic status; GRS = genetic risk score; BMI= body mass index; wGRS = weighted genetic risk score. Adjusted regression analysis was used to evaluate the association where the model was adjusted for SES, sex, age, BMI and GRS (**A**) and with wGRS (**B**).

**Table 4:** Association of smoking behaviours with SES and other variables.

Smoking Behaviours		Model V			Model VI		
		OR	95% CI	*p*-Value	OR	95% CI	*p*-Value
Former Smoker	GRSs	1.033	0.702–1.521	0.870	1.011	0.762–1.520	0.675
	SES = upper lower	1.005	1.000–1.088	**<0.001**	1.335	1.296–1.951	**<0.001**
	SES = lower middle	1.059	1.042–1.221	**<0.001**	1.482	1.045–1.700	**<0.001**
	SES = upper middle	1.357	1.350–1.753	**<0.001**	1.561	1.451–1. 601	**<0.001**
	Age	1.274	0.887–1.83	0.190	1.275	0.885–1.826	0.194
	BMI	0.847	0.535–1.342	0.480	0.847	0.534–1.341	0.477
	[Sex = Male]	0.538	0.173–1.677	0.285	0.539	0.173–1.677	0.285
	[Population = HG]	0.287	0.091–1.403	**0.014**	0.285	0.272–1.265	**0.015**
Moderate Smoker	GRSs	1.027	0.865–1.218	0.764	1.025	0.817–1.106	0.514
	SES = upper lower	1.991	1.885–2.557	**<0.001**	1.274	1.111–1.278	**<0.001**
	SES = lower middle	1.253	1.152–1.778	**<0.001**	1.095	1.091–1.619	**<0.001**
	SES = upper middle	0.385	0.285–0.850	**<0.001**	0.625	0.625–0.962	**<0.001**
	Age	1.063	0.904–1.250	0.457	0.904	0.907–1.254	0.436
	BMI	0.746	0.604–0.922	**0.007**	0.604	0.604–0.921	**0.006**
	[Sex = Male]	0.853	0.543–1.340	0.490	0.854	0.543–1.342	0.493
	[Population = HG]	0.383	0.292–4.023	**<0.001**	0.543	0.236–4.130	**<0.001**
Heavy Smoker	GRSs	1.027	0.88–1.199	0.737	1.045	0.911–1.199	0.527
	SES = upper lower	1.979	1.155–2.177	**<0.001**	1.662	1.228–1.688	**<0.001**
	SES = lower middle	1.113	1.098–1.855	**<0.001**	1.892	1.706–1.905	**<0.001**
	SES = upper middle	0.885	0.655–1.022	**<0.001**	0.976	0.679–0.987	**<0.001**
	Age	0.983	0.85–1.1370	0.816	0.981	0.85–1.1370	0.818
	BMI	0.734	0.609–0.884	**0.001**	0.733	0.608–0.883	**0.001**
	[Sex = Male]	1.622	1.097–2.400	**0.015**	1.623	1.095–2.397	**0.016**
	[Population = HG]	0.151	0.109–4.996	**<0.001**	0.151	0.109–4.934	**<0.001**

Bold font highlights significant results. Never smoker was set as a reference. SES = socioeconomic status; GRSs = genetic risk scores; BMI = body mass index; HG = Hungarian General. Model V = using genetic risk score (GRS) with SES, BMI, age, sex, and population as independent variables. Model VI = using weighted genetic risk score (wGRS) with SES, BMI, age, sex, and population as independent variables. SES = upper was set as a reference category; the lower category of SES was removed from the table, as it was considered as a redundant because no single HG subject was indicated in this category. The R^2^ of the association was 0.176.

## Data Availability

No additional data available.

## Supplementary Materials

The following are available online at https://www.mdpi.com/1660-4601/18/6/3206/s1. Table S1: Hardy-Weinberg equilibrium for Hungarian Roma; Table S2: Smoking status among both populations based on gender; Table S3: Differences in risk allele frequencies between study populations; Table S4: Risk allele differences for males of both populations; Table S5: Risk allele differences for females of both populations; Table S6: Genotype by smoking behaviours in general population; Table S7: Genotype by smoking behaviours in the Roma population; Table S8: GRSs and Smoking behaviours; Table S9: Predicted model on cigarette per day in both populations; Table S10: Regression model on age-imitation of smoking in both populations; Table S11: Socioeconomic status according to smoking status in both population; Figure S1: Cigarette per day (CPD) by genotype in the whole study sample; Figure S2: Cigarette per day (CPD) by genotype among HG (a1,2), (b1,2) among HR; Figure S3: Age at smoking initiation by genotype; Figure S4: Age at smoking initiation by genotypes among HG (a1,2), (b1,2) among HR; Figure S5: Frequency distributions of GRS and wGRS based on populations.
