# Supplementary material for: Roma Socioeconomic Status Has a Higher Impact on Smoking Behaviour than Genetic Susceptibility
Source: Int J Environ Res Public Health. 2021 Mar 19;18(6):3206. doi: 10.3390/ijerph18063206 (PMC8003628; doi:10.3390/ijerph18063206)
Supplement: Supplementary file 1 [file ijerph-18-03206-s001.pdf]

Supplementary Materials for the manuscript entitled

Roma Socioeconomic Status Has Higher Impact On Smoking Behaviour Than Genetic Susceptibility

Mohammed A. Merzah MPH[1,2], Zsigmond Kósa PhD [3], János Sándor PhD [1], Shewaye Natae MPH[1,2], Péter Pikó MSc[5], Róza Ádány DSc [1,5], Szilvia Fiatal PhD[1]

[1]*Department of Public Health and Epidemiology, Faculty of Medicine, University of Debrecen, 4032 Debrecen, Hungary;*
[2]*Doctoral School of Health Sciences, University of Debrecen, 4032, Debrecen, Hungary;*
[3]*Department of Health Methodology and Public Health, Faculty of Health, University of Debrecen,4400 Nyíregyháza, Hungary;*
[5]*MTA-DE Public Health Research Group, University of Debrecen,4032 Debrecen, Hungary*

Table 1: Hardy- Weinberg equilibrium for Hungarian Roma

CHR	SNP	Gene	A1	A2	GENO	p-value
2	rs10490162	NRXN1	C	T	3/66/327	1
4	rs3762611	GABRB1	A	G	21/105/272	**0.0186**
5	rs2673931	TRPC7	C	T	80/207/112	0.4204
10	rs4142041	CTNNA3	G	A	50/176/171	0.6586
15	rs2036534	AGPHD1	C	T	25/143/230	0.6823
15	rs16969968	CHRNA5	A	G	48/166/184	0.2586
15	rs578776	CHRNA3	A	G	74/171/148	0.05945
21	rs6517442	KCNJ6	C	T	47/186/166	0.7415
23	rs2235186	MAOA	A	G	43/147/105	0.5423

Table 2: Smoking status among both populations based on gender.

Gender	HR		HG		OR	95% *CI*
	SM %	NSM %	SM %	NSM %		
Males	16.5	9.8	15.8	29.3	1.892	1.588 - 2.255
Females	48.7	25.0	17.0	37.9	2.054	1.475 - 2.860

SM= smokers; NSM= non-smokers

Table 3: Differences in risk allele frequencies between study populations.

SNP	Gene	Risk Allele	Frequency HR	HG	χ2	p-value	OR	ADJ-*p**
rs10490162	NRXN1	T	0.91	0.89	1.518	0.218	1.232	0.249
**rs2673931**	**TRPC7**	**T**	**0.54**	**0.62**	**10.240**	**0.001**	**0.720**	**0.005**
rs4142041	CTNNA3	G	0.35	0.39	2.366	0.124	0.851	0.165
rs2036534	AGPHD1	T	0.77	0.80	3.046	0.081	0.807	0.129
rs16969968	CHRNA5	A	0.33	0.36	1.064	0.302	0.896	0.302
**rs578776**	**CHRNA3**	**G**	**0.60**	**0.74**	**35.270**	**<0.0001**	**0.524**	**<0.0001**
**rs6517442**	**KCNJ6**	**C**	**0.35**	**0.29**	**5.681**	**0.017**	**1.295**	**0.030**
**rs2235186**	**MAOA**	**A**	**0.40**	**0.32**	**9.239**	**0.002**	**1.429**	**0.006**

Bold font highlights significant differences; * adjustable p-value

Table 4: Risk allele differences for males of both populations.

SNP	Risk Allele	Frequency HR	HG	χ2	p-value	OR	ADJ-*p*[1]
rs10490162	T	0.90	0.91	0.221	0.638	0.871	0.729
rs2673931	T	0.50	0.62	7.553	**0.006**	0.613	**0.048**
rs4142041	G	0.34	0.40	2.040	0.153	0.768	0.245
rs2036534	T	0.75	0.78	0.457	0.499	0.870	0.666
rs16969968	A	0.34	0.34	0.004	0.949	0.988	0.949
rs578776	G	0.62	0.71	5.354	**0.021**	0.650	0.055
rs6517442	C	0.39	0.30	4.415	**0.036**	1.476	0.071
rs2235186	A	0.44	0.30	5.877	**0.015**	1.882	0.055

Bold fonts highlight significant differences. Legend: [1] adjusted p-value.

Table 5: Risk allele differences for females of both populations.

SNP	Risk Allele	Frequency		χ2	p-value	OR	ADJ-*p*[1]
		HR	HG				
rs10490162	T	0.91	0.88	3.759	0.053	1.498	0.084
rs2673931	T	0.55	0.62	4.493	**0.034**	0.760	0.084
rs4142041	G	0.36	0.39	0.767	0.381	0.891	0381
rs2036534	T	0.77	0.82	4.113	**0.043**	0.723	0.084
rs16969968	A	0.33	0.37	1.621	0.203	0.844	0.232
rs578776	G	0.59	0.76	32.280	**<0.0001**	0.452	**<0.0001**
rs6517442	C	0.34	0.29	2.534	0.111	1.245	0.149
rs2235186	A	0.39	0.33	4.718	**0.030**	1.336	0.084

Bold fonts highlight significant differences. Legend: [1] adjusted p-value.

Table 6: Genotype by smoking behaviours in general population (n=412)

SNPs	Genotype	Smoking Behaviors				p-value
		HSM[a]	MSM[b]	FSM[c]	NSM[d]	
rs10490162-T	C C	0.0	0.5	0.0	1.0	0.455
	T C	1.2	2.2	0.0	15.6	
	T T	10.8	11.2	1.7	55.7	
rs16969968-A	A A	1.7	1.0	0.0	8.0	0.094
	G A	7.6	7.6	1.2	32.9	
	G G	2.7	5.4	0.5	31.5	
rs2036534-T	C C	0.0	0.2	0.0	3.2	0.144
	C T	2.9	3.9	0.7	25.7	
	T T	9.0	9.5	1.0	43.8	
**rs2235186-A**	A A	3.9	2.2	0.0	12.5	**0.001**
	A G	2.0	4.2	1.5	18.6	
	G G	5.9	7.6	0.2	41.6	
rs2673931-T	C C	1.0	3.2	0.2	9.6	0.144
	C T	7.6	5.1	1.0	34.6	
	T T	3.2	5.6	0.5	28.4	
rs4142041-G	A A	5.9	5.2	0.2	23.7	0.476
	A G	4.7	6.9	1.2	39.0	
	G G	1.5	2.0	0.2	9.4	
rs578776-G	A A	0.2	0.5	0.0	5.7	0.546
	G A	4.7	5.5	1.2	28.0	
	G G	7.2	7.9	0.5	38.5	
rs6517442-C	C C	1.0	1.7	0.0	5.9	0.052
	T C	3.7	5.1	0.0	32.7	
	T T	7.3	7.1	1.7	33.9	

Legend: [a] Heavy Smoker, [b] Moderate Smoker, [c] Former Smoker,[d] Never smoker. Bold font means statistically significant at (0.001) level. Risk allele is written besides each SNP.

Table 7: Genotype by smoking behaviours in the Roma population (n=402)

SNPs	Genotype	Smoking Behaviors				p-value
		HSM[a]	MSM[b]	FSM[c]	NSM[d]	
rs10490162-T	C C	0.5	0.3	0.0	0.0	0.763
	T C	6.8	2.8	0.8	6.3	
	T T	30.8	16.2	2.8	32.8	
rs16969968-A	A A	3.5	2.3	0.3	6.0	0.407
	G A	15.8	8.8	2.3	14.8	
	G G	19.1	8.0	1.3	17.8	
rs2036534-T	C C	3.0	0.8	0.0	2.5	0.880
	C T	14.3	7.3	1.0	13.3	
	T T	20.9	11.1	2.8	23.1	
rs2235186-A	A A	10.5	2.8	1.0	7.8	0.356
	A G	14.3	8.0	1.5	13.3	
	G G	13.5	8.3	1.3	17.8	
rs2673931-T	C C	8.3	3.5	1.0	7.3	0.891
	C T	18.5	10.0	2.0	21.3	
	T T	11.5	5.5	0.8	10.3	
rs4142041-G	A A	15.9	8.3	2.3	16.6	0.418
	A G	16.1	9.6	1.0	17.6	
	G G	6.0	1.0	0.5	5.0	
rs578776-G	A A	7.1	2.5	0.8	8.4	0.533
	G A	18.3	8.7	1.0	15.5	
	G G	13.0	7.6	1.8	15.3	
rs6517442-C	C C	4.5	1.3	0.5	5.5	0.439
	T C	17.5	10.0	2.5	16.5	
	T T	16.3	7.8	0.8	16.8	

Legend: [a] Heavy Smoker, [b] Moderate Smoker, [c] Former Smoker, [d] Never smoker. Risk allele is written besides each SNP.

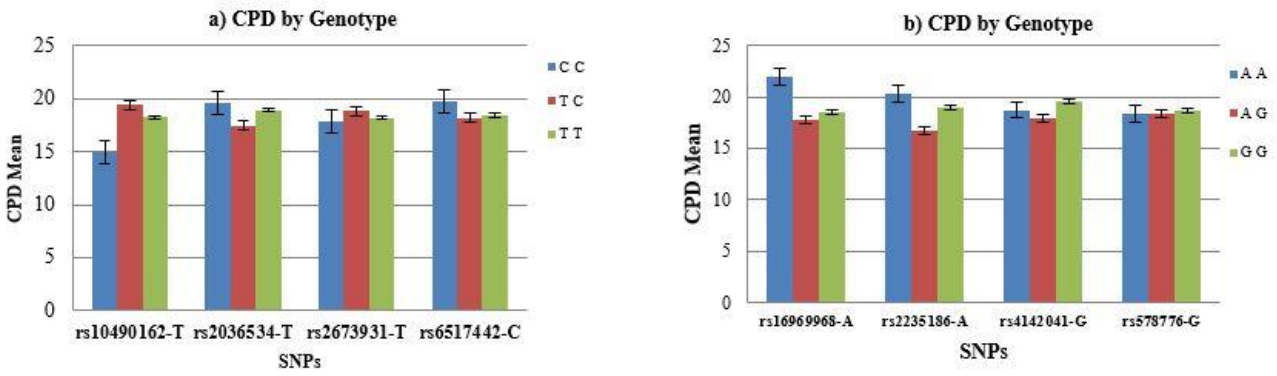

Figure 1: Cigarette per day (CPD) by genotype in the whole study sample. Risk allele is written beside each SNP

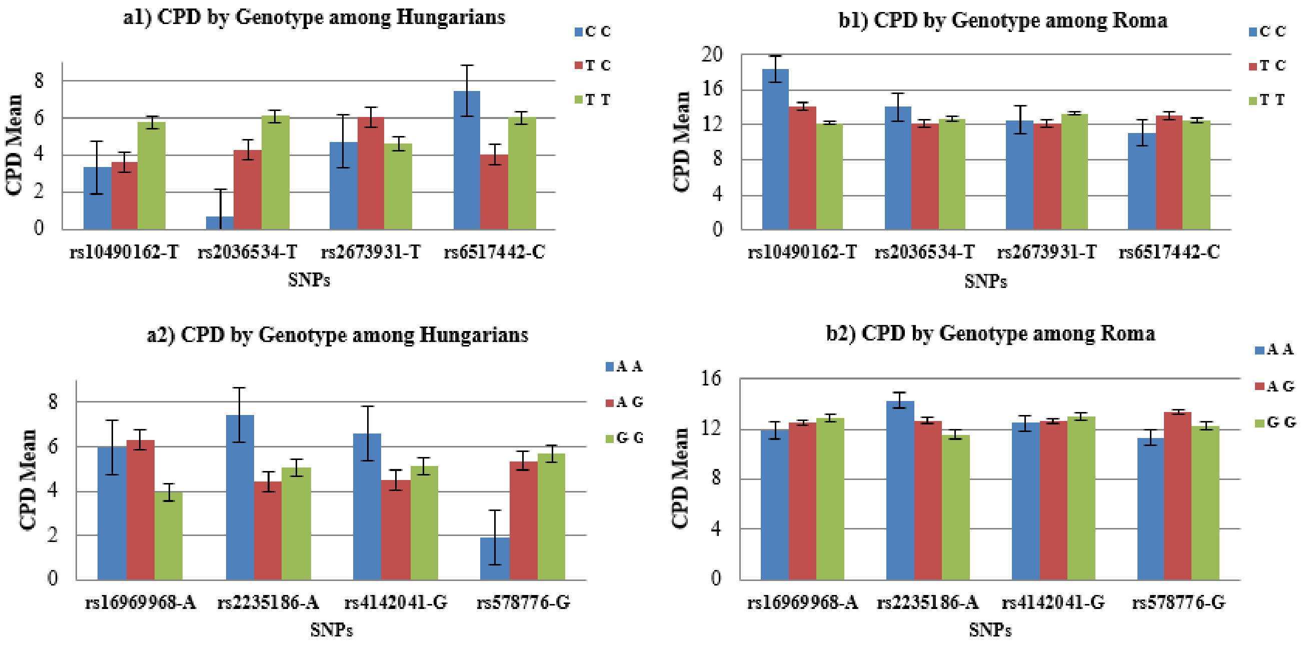

Figure 2: Cigarette per day (CPD) by genotype among HG (a1,2), (b1,2) among HR. Risk allele is written beside each SNP

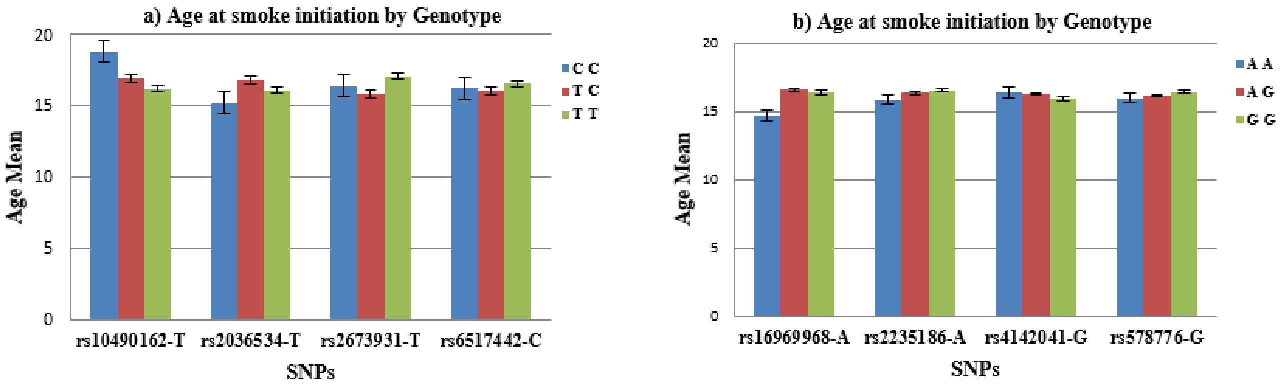

Figure 3: Age at smoking initiation by genotype. Risk allele is written beside each SNP

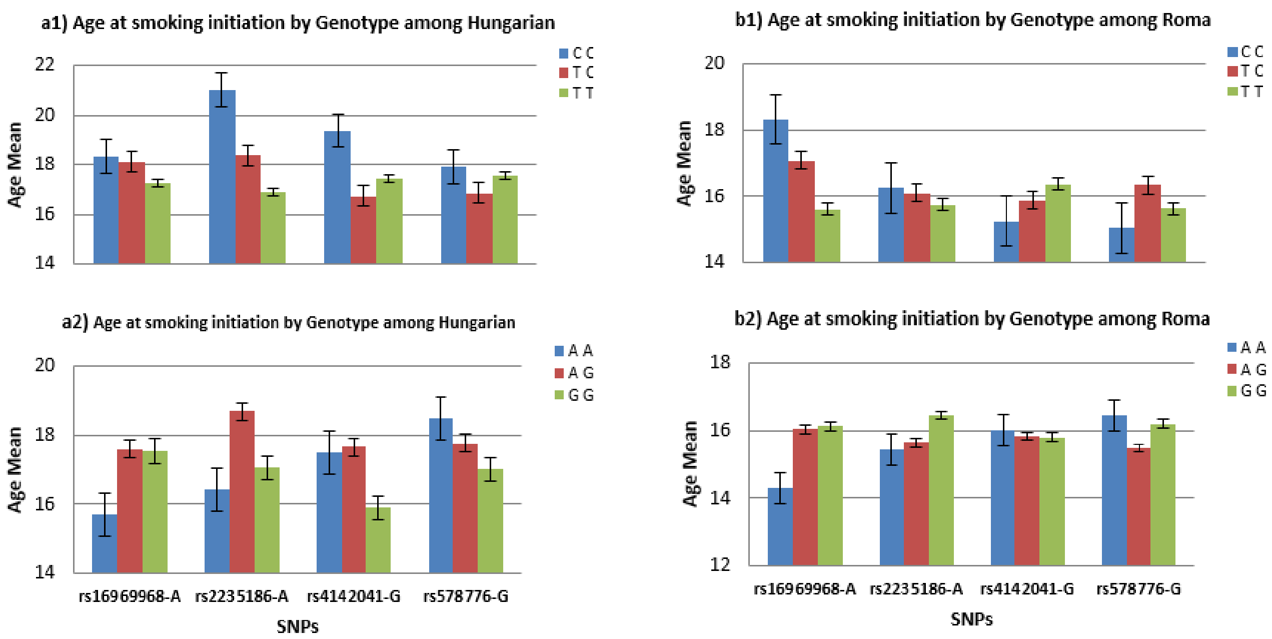

Figure4: Age at smoking initiation by genotypes among HG (a1,2), (b1,2) among HR. Risk allele is written beside each SNP

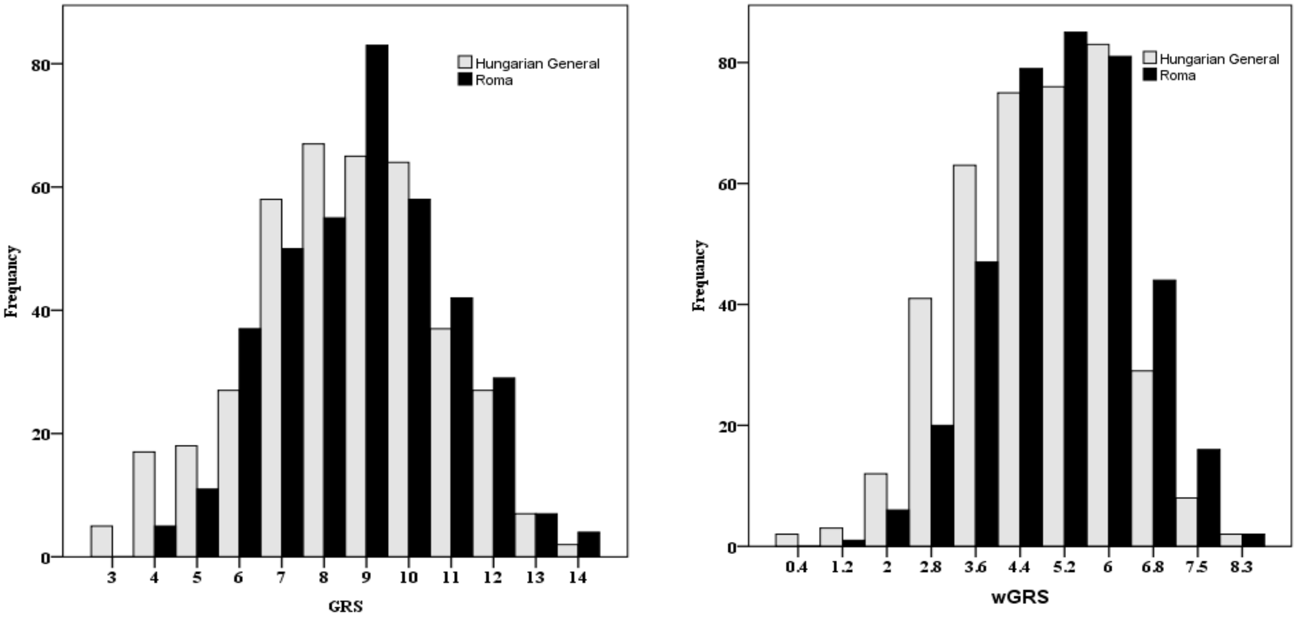

Figure5: Frequency distributions of GRS and wGRS based on populations

Table 8: GRSs and Smoking behaviours

Smoking Behaviors	Hungarian Roma (HR)						Hungarian General (HG)					
	GRS	OR	95% *CI*	wGRS	OR	95% *CI*	GRS	OR	95% *CI*	wGRS	OR	95% *CI*
Heavy Smokers	0.61	1.07	0.98-1.15	0.57	0.94	0.86-1.42	0.52	1.02	0.91-1.2	0.65	1.34	0.32-0.97
Other smokers	0.57			0.61			0.51			0.48		

Table 9: Predicted model on cigarette per day in both populations.

	Cigarette per day (CPD)							
	Model I[a]				Model I[b]			
	β	Standardized β	*t*	p-value	β	Standardized β	*t*	p-value
GRSs	-0.040	-0.007	-0.215	0.829	0.064	0.007	0.218	0.827
Population	7.665	0.330	9.275	**0.000**	7.625	0.329	9.172	**0.000**
SES	-0.055	-0.016	-0.461	0.645	-0.056	-0.016	-0.467	0.641
Gender	-2.452	-0.101	-2.927	**0.004**	-2.448	-0.101	-2.923	**0.004**
Age	-0.297	-0.033	-0.951	0.342	-0.302	-0.034	-0.969	0.333
BMI	-1.176	-0.101	-2.901	**0.004**	-1.175	-0.101	-2.898	**0.004**

Bold font highlights significant result. R^2=.113 Legend: a= CPD was set as a dependent variable while GRS, population, SES, gender, age and BMI were set as independent variables; b=CPD was set as a dependent variable while wGRS, population, SES, gender, age and BMI were set as independent variables. Hungarian General was set as a reference for population. R^2= 0.051

Table10: Regression model on age-imitation of smoking in both populations.

	Age- initiation of smoking							
	Model II[a]				Model II[b]			
	β	Standardized β	*t*	p-value	β	Standardized β	*t*	p-value
GRSs	0.068	0.016	0.462	0.645	0.028	0.004	0.121	0.904
Population	-4.054	-0.231	-6.170	**0.000**	-4.069	-0.232	-6.155	**0.000**
SES	0.019	0.007	0.204	0.839	0.020	0.008	0.212	0.832
Gender	0.031	0.002	0.047	0.963	0.030	0.002	0.045	0.964
Age	0.201	0.030	0.814	0.416	0.203	0.030	0.825	0.410
BMI	-0.702	-0.079	-2.186	**0.029**	-0.701	-0.079	-2.181	**0.030**

Bold font highlights significant result. Legend: a= Age-initiation of smoking was set as a dependent variable while GRS, population, SES, gender, age and BMI were set as independent variables; b= Age-initiation of smoking was set as a dependent variable while wGRS, population, SES, gender, age and BMI were set as independent variables. Hungarian General was set as a reference for population. R^2= 0.051

Table 11: Socioeconomic status according to smoking status in both population.

Socioeconomic	Hungarian Roma (HR)				Hungarian General (HG)			
	SM	NSM	χ^2	p-value	SM	NSM	χ^2	p-value
Lower	21(5.2)	0(0)	8.076	**0.001**	0(0)	0(0)	15.93	**0.005**
Upper lower	71(17.7)	35(8.7)			25(6.1)	44(10.7)		
Lower middle	121(30.1)	68(16.9)			67(16.3)	108(26.2)		
Upper middle	49(12.2)	37(9.2)			42(10.2)	108(26.2)		
Upper	0(0)	0(0)			1(0.2)	17(4.1)		

Bold font highlights significant results.
